# Controlling Indoor Air Pollution from Moxibustion

**DOI:** 10.3390/ijerph13060612

**Published:** 2016-06-20

**Authors:** Chung-Yen Lu, Sy-Yuan Kang, Shu-Hui Liu, Cheng-Wei Mai, Chao-Heng Tseng

**Affiliations:** 1 School of Post-Baccalaureate Chinese Medicine, China Medical University, Taichung 404, Taiwan; u100030082@cmu.edu.tw; 2 Department of Sport and Health Management, Da-Yeh University, Changhua 515, Taiwan; 3 Institute of Environmental Engineering and Management, National Taipei University of Technology, Taipei 106, Taiwan; jeffrey.kang@msa.hinet.net (S.-Y.K.); t6679010@ntut.edu.tw (C.-W.M.); 4 Department of Health Care and Social Work, Yu-Da University of Science and Technology, Miaoli 361, Taiwan; liush@ydu.edu.tw; 5 Department of Public Health, China Medical University, Taichung 404, Taiwan

**Keywords:** indoor air quality, moxibustion, moxa wool, local exhaust ventilation, air cleaner

## Abstract

Indoor air quality (IAQ) control of hospitals plays a critical role in protecting both hospital staffs and patients, particularly those who are highly susceptible to the adverse effects of indoor noxious hazards. However, moxibustion in outpatient departments (OPDs) of traditional Chinese medicine (TCM) may be a source of indoor air pollution in hospitals. Some studies have investigated indoor air pollution during moxibustion in Chinese medicine clinics (CMCs) and moxibustion rooms, demonstrating elevated air pollutants that pose a threat to the health of medical staff and patients. Our study investigated the indoor air pollutants of indoor carbon dioxide (CO_2_), carbon monoxide (CO), formaldehyde (HCHO), total volatile organic compounds (TVOCs), airborne particulate matter with a diameter of ≤10 µm (PM_10_) and ≤2.5 µm (PM_2.5_) during moxibustion in an acupuncture and moxibustion room of the OPD in a hospital in Taipei. To evaluate the different control strategies for indoor air pollution from moxibution, a comparison of air pollutants during moxibution among the methods of using alternative old moxa wools, local exhaust ventilation and an air cleaner was conducted. In this study, burning alternative old moxa wools for moxibustion obviously reduced all gaseous pollutants except for aerosols comparing burning fresh moxa wools. Using local exhaust ventilation reduced most of the aerosols after burning moxa. We also found that using an air cleaner was inefficient for controlling indoor air pollutants, particularly gaseous pollutants. Therefore, combining replacing alternative old moxa wools and local exhaust ventilation could be a suitable design for controlling indoor air pollution during moxibustion therapy.

## 1. Introduction

Hospital indoor air pollution has become a public concern in preventing patients and health care workers from contracting hospital-acquired infections and occupational diseases. Inadequate indoor environments in hospitals are associated with specific building materials, air conditioning systems, ventilation rates, and human behaviors [1]. Indoor air pollution can cause oxidative stress [2,3] and outbreaks of building-related illness such as dry throat, irritability, tiredness, dizziness, and other symptoms [4]. Controlling the indoor air quality (IAQ) of hospitals plays a critical role in protecting both hospital staffs and patients, particularly those who are highly susceptible to the adverse effects of indoor noxious hazards. Hence, the Environmental Protection Administration of Taiwan established the Indoor Air Quality Management Act and assisted hospitals in the first stage of the law’s implementation to ensure that they satisfied the stipulated standards regarding the following indoor pollutants: carbon dioxide (CO); carbon monoxide (CO_2_); formaldehyde (HCHO); total volatile organic compounds (TVOCs); ozone; airborne particulate matter with a particle diameter in the 2.5–10 µm range (PM_10_) and of ≤2.5 µm (PM_2.5_); bacteria; and fungi [5]. However, people in Taiwan have overlooked one crucial public venue that might be a source of indoor air pollutants—Chinese medicine clinics (CMCs) and outpatient departments (OPDs) at traditional Chinese medicine (TCM) in hospitals. In Taiwan, the National Health Insurance program (NHI) has provided healthcare coverage to more than 99% of the population entire 23 million Taiwan’s residents by 2011 [6], hence, there are lots of people to accept TCM, including Chinese herbal medicines, acupuncture, tui na, and moxibustion, for treating or preventing diseases.

Moxibustion is a kind of external treatment based on the theory of TCM applying the thermal energy of burning moxa wools—in a session that typically lasts 15–30 min—to stimulate certain acupuncture points and thus help dredge meridians, regulate internal systems of the body and prevent or cure diseases [7]. Moxibustion uses moxa produced from dried *Artemisia* (mugwort) herb species after aging, grinding up to a fluff and further processing into a cigar-shaped stick. The cigar-shaped moxa wools could be indirectly used with acupuncture needles or burnt close to the patient's skin for local heat stimulus and pain control. Moxibustion are jiǔ, kyū and tteum, respectively. Fresh moxa wools are made up by fresh mugwort. Conversely, old moxa wools are made from aged mugwort, stored for three or more years and therefore more expensive. Hence, physicians commonly use fresh moxa wools in clinical treatment of patients.

Medical staffs and some patients may be exposed to elevated concentrations of moxa smoke. Some studies have reported that moxa smoke had disinfective antibacterial properties, while others demonstrated adverse reactions whose symptoms were similar to those of hay fever [8]. Mo *et al.* reported that elevated concentrations of monoaromatic hydrocarbons, HCHO, and polycyclic aromatic hydrocarbons (535.2, 157.9, and 12.86 ppm, respectively) were notable health risks in two moxibustion rooms [9]. In addition, Hsu *et al.* investigated indoor air aldehyde levels during moxibustion in a CMC and demonstrated mean HCHO and acetaldehyde concentrations of 654 and 4230 μg/m^3^, respectively, which pose a threat to the health of medical staff and patients [10]. Although both Mo and Hsi demonstrated that air quality can be improved by ventilation, many previously ventilated buildings including hospitals, have been replaced by more energy-efficient and airtight buildings since the mid-1970s for saving energy and this may be the cause of building-related symptoms [11]. Controlling air pollution from moxibustion therapy in hospitals is crucial, however, few studies have been conducted to investigate methods for improving air pollution from burning moxa wools. Thus, this study was conducted to compare the variances in indoor air pollutants released from moxibustion from using different improvement methods and identify an effective control strategy.

## 2. Methods

**Figure 1:**
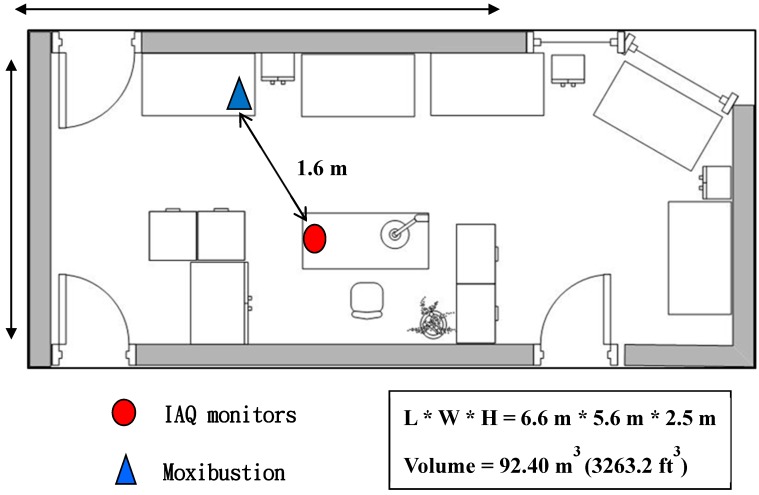
Diagram of the acupuncture and moxibustion room of the hospital.

Permits to conduct this study were obtained from a hospital in Taipei City, Taiwan. IAQ was evaluated in a test room, the acupuncture and moxibustion room of the Chinese medicine OPD in the hospital, which was equipped with central heating ventilation and air-conditioning (HVAC) systems. Air exchange rates were estimated by calculating the hourly ventilation rate divided by indoor volume and ranged from 4 to 10 air changes per hour (ACH) depending on the differences between indoor and outdoor temperatures. Indoor temperature was maintained at 24 ± 1 °C. Figure 1 displays the spatial layout of the room, with a volume of 92.40 m^3^, which is located in a closed space with three exits. The doors are kept closed during moxibustion therapy to protect patients’ privacy. There are five therapy beds, a desk, a chair, three sets of cabinets and three sets of bedside cabinets inside the room. For the purposes of this study there was only a patient, a medical staff member and an air sampling device in the room, and the doors of the three exits were closed and the HVAC system was not in use during the air-sampling period.

### 2.1. Pollutant Emission Monitoring

#### 2.1.1. Indoor Air Quality Monitoring

In this study, the diameter of the moxa wools was 18 mm and the length was 200 mm. Temporal variations of IAQ inside the acupuncture and moxibustion room were measured at the background stage without burning a new moxa wool for 10 min, at the moxibustion stage by burning a new moxa wool for 15 min, and during a decay period of 20 min after the new moxa wool had finished combusting. To simulate the breathing zone of seated healthcare workers and avoid interference from indoor ventilation, on-site measurement of air quality inside the acupuncture and moxibustion room was performed for air temperature, relative humidity, and concentrations of CO, CO_2_, HCHO, TVOCs, PM_10_ and PM_2.5_ at 1.2 m height and respectively 1.2, 4.0 and 4.8 m far from the three exits as well as at a 1.6 m distance from the moxibustion source on the therapy bed. CO and CO_2_ were measured with an AirBoxx monitor (KD Airboxx, KD Engineering Inc., Blaine, WA, USA) using an electrochemical technique with a detection range of 0–200 ppm and a nondispersive infrared technique with a detectable range of 0–10,000 ppm, respectively. Calibration was conducted using 5000 ppm CO_2_ for the span gas and nitrogen for the zero gas before sampling. Temperature and humidity were also measured with the AirBoxx monitor by using a thermistor operating in a range of 0 °C–50 °C and an interchangeable capacitor with a detectable range of 5%–95%, respectively. TVOCs were measured with a photoionization detector (IAQRAE PGM-5210, RAE Systems, Sunnyvale, CA, USA) with a range of 0–9.99 ppm and built-in correction for 102 VOC gases. Calibration was conducted using 50,000 ppb isobutylene for the span gas and pure (hydrocarbon-free) air for the zero gas before sampling. The PM_10_ and PM_2.5_ levels were assessed using a portable dust monitor (Aerocet-531; Met One Instrument, Inc., Grants Pass, OR, USA) based on a laser diode technique with a range of 0.01–10 mg/m^3^. Calibration was conducted using a filter for a zero count test before sampling, and the device was sent to the manufacturer to replace the internal filter as well as calibrate the sensor using calibrating particle counters, and NIST traceable standards, every two years. HCHO was measured with a formaldemeter (PPM Formaldemeter™ htV, PPM Technology Ltd., Caernarfon, Wales, UK) using an electrochemical technique with a detection range of 0–10 ppm. Calibration was conducted by comparing with the measured values of an atmospheric monitoring station every season and calibrated to ISO/IEC17025 by the manufacturer every year. The indoor air was monitored in the acupuncture and moxibustion room for 45 min (10 min for background, 15 min for moxibustion, and 20 min for decay of the air pollution). Indoor air was well-mixed and conditioned by an electric fan during the sampling period. Samples were collected in triplicate to ensure the sampling reliability and data were presented as the average of three samples.

#### 2.1.2. Qualitative and Quantitative Analyses

For qualitative and quantitative analyses of air pollutants emitted during moxibustion of fresh and old wools, emissions were sampled using a personal sampling pump coupled to an active carbon tube (50/100 mg) with a flow rate of 202.5 mL/min for 1 h in an empty test room with a volume of 79.40 m^3^. For the qualitative analysis, a gas chromatograph (GC) model 6890N (Agilent Technologies, Santa Clara, CA, USA) equipped with an Agilent Technologies model 5973N mass spectrometry detector (MS) was used. Separations were carried out on an Abel Bonded AB-624 capillary column. Injector temperature was set to 280 °C. Helium (constant pressure) was employed as carrier gas at a flow rate of 1.0 mL/min. The oven temperature for the analysis was initially programmed at 35 °C and heated at a rate of 15 °C/min till 220 °C. For the quantitative analysis, the compounds found in both the emissions of fresh and old moxa wools were determined by using a model GC-14B GC from Shimadzu (Kyoto, Japan) equipped with a thermal conductivity detector (TCD) and a flame ionization detector (FID). Separations were carried out on a Shimadzu DB-1 capillary column, stationary phase of 5% phenyl–95% dimethylsiloxane (30 m × 0.53 mm i.d., 1.5 µm film thickness) and nitrogen as carrier gas (3.8 mL/min). Injector temperature was set to 280 °C. The detector temperature was of 250 °C for all analyses. The column temperature programming was adjusted initially at 50 °C and heated at a rate of 8 °C/min till 100 °C. The chromatographic systems were thoroughly cleaned with acetonitrile injections in the same analytical conditions after each analysis. Simultaneously, TVOCs were continuously monitored using the IAQRAE PGM-5210 photoionization detector.

### 2.2. Indoor Air Pollution Control Strategies

#### 2.2.1. Alternative Old Moxa Wool

We compared the indoor air pollutant emissions from burning fresh and old moxa wools. Three components of the physicochemical properties (moisture, ash, and volatile matter) and burning rates of moxa wools were analyzed in triplicate. Moisture was determined by drying moxa samples at 105 °C for 2 h. Weight loss was expressed as a percentage of the initial weight of the samples. Ash was weighed from the residue obtained after complete combustion of moxa samples at 800 ± 50 °C for 3 h. Ash weight was expressed as a percentage of the initial weight of the samples. Volatile matter was determined by deducting the moisture and ash weight from the initial weight of samples. Volatile matter weight was also expressed as a percentage of the initial weight of samples. Burning rate was determined by deducting the residual weight from the initial weight of samples and then dividing by the moxibustion period (15 min).

#### 2.2.2. Local Exhaust Ventilation

**Figure 2:**
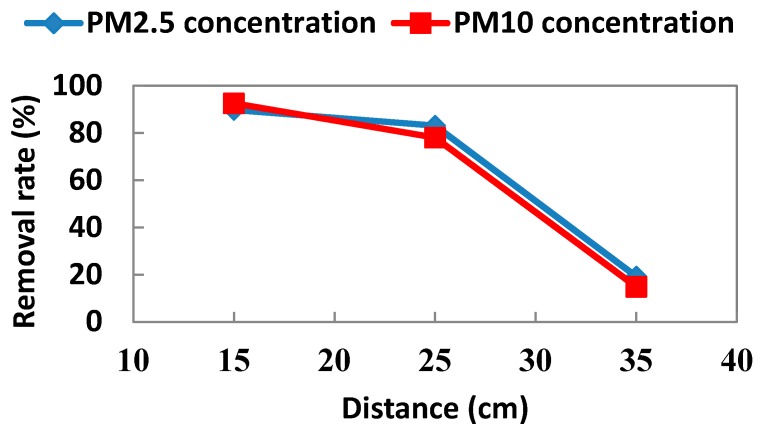
Comparison among the measurements of indoor air particulates from moxibustion emissions inside the acupuncture and moxibustion room with local exhaust ventilation at the distance of 15, 25 and 35 cm far from the combustion sources.

A cyclone dust collector with an air velocity of 0.4 m/s and air flow of 0.19 m^3^/min made up of a semicircle hood, a movable extraction arm, a ventilator and a tube, was selected to remove local combusting contaminants to the outdoors. The dust collector was placed 25 cm from the combustion of fresh moxa wools due to the acceptable removal rate (83.69% for PM_2.5_ and 78.04% for PM_10_, respectively) in our pre-test, however less influence was noted on medical therapy by avoiding the rapid combustion of moxa wools (Figure 2).

#### 2.2.3. Air Cleaner

A carbon air cleaner (CAL-ZA12A, SAMPO, Taipei, Taiwan) with a high-efficiency particulate arrestance (HEPA) air filter combined with a titanium oxide ultraviolet light photocatalyst and an air ion generator was selected to place in the middle of the test room to remove air pollutants from the moxibustion. The power, air velocity and air flow of the air cleaner were set to 50.0 watt, 3.6 m/s as well as 4.15 m^3^/min, respectively.

## 3. Results and Discussion

### 3.1. Pollutant Emission Monitoring

#### 3.1.1. CO and CO_2_ Concentrations

**Figure 3:**
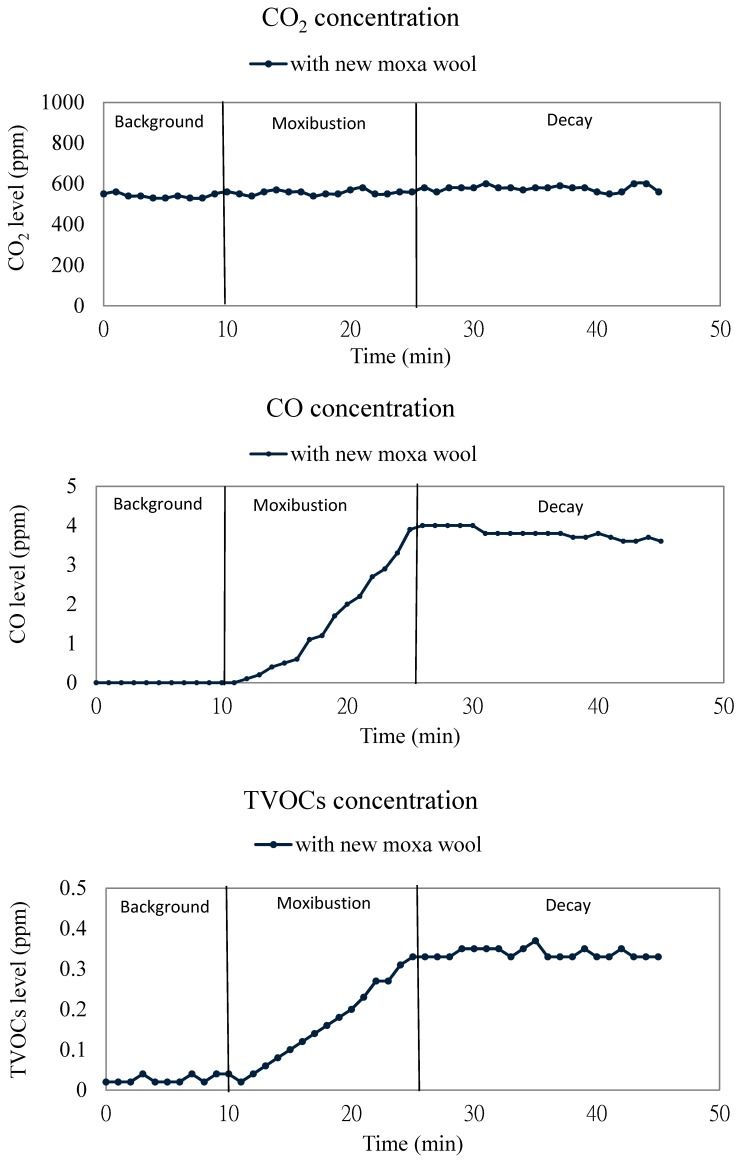

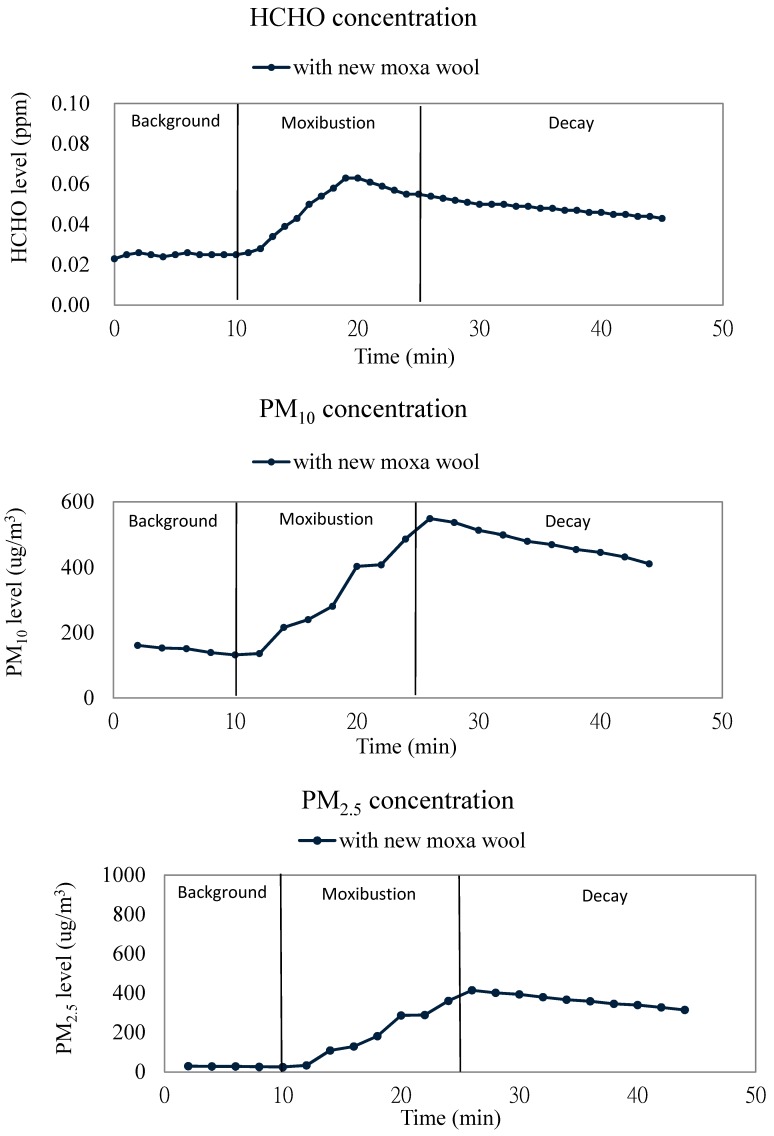
Continuous measurements of Indoor air pollutants from moxibustion emission of a new moxa inside the acupuncture and moxibustion room.

The presented data were the means of samples measured in triplicate (Figure 3). Continuous measurements demonstrate that CO levels inside the acupuncture and moxibustion room was closely associated with the moxibustion. When the moxibustion was started, CO concentrations sharply increased from a background level of <0.1 ppm to the maximum concentration of 4.0 ppm. The average of the increased concentration during the moxibustion stage was 2.8 ppm. The moxibustion likely produced a large amount of CO because of incomplete combustion. CO_2_ concentrations increased slightly from 542 ppm at the background stage to 569 ppm during the moxibustion stage. CO_2_ is a natural air component whose source is usually due to the occupants’ exhalation. CO_2_ is a proper indicator of adequate and efficiency ventilation and is related to indoor air pollutants’ accumulation [12]. However, the variance of CO_2_ is not similar to that of other pollutants in our study.

#### 3.1.2. TVOCs Concentration

Mean concentrations of TVOCs increased inside the acupuncture and moxibustion room during moxibustion. At the moxibustion stage, the mean levels of TOVCs increased from the background level of 0.03 ppm to the maximum concentration of 0.37 ppm. The average concentration increase at the moxibustion stage was 0.28 ppm. Mo *et al*. reported that the concentrations of monoaromatic hydrocarbons, HCHO, and polycyclic aromatic hydrocarbons in two moxibustion rooms were 535.2, 157.9, and 12.86 μg/m^3^, respectively [9]. Comparing the TVOC concentration results obtained by different studies was difficult because of the differences in sampling and analytical methods. In the present study, measured TVOC concentrations produced by burned moxa wools were in the range of 0.02–0.37 ppm. Although these TVOC concentrations were lower than the standards stipulated in Taiwan’s Indoor Air Quality Act of 0.56 ppm (1-h average), thus may have been a cumulative effect from a real working day in which there are approximately 20 or more patients per 3.5 h undergoing moxibustion therapies and several moxa wools were used with each one to stimulate different acupuncture points. During moxibustion, mean TOVC concentrations increased to more than 12-fold the background mean level. Lu’s studies showed that office worker’s building-related symptoms and oxidative stress indicated by urinary 8-hydroxydeoxyguanosine (8-OHdG) was positively associated with TVOC concentrations in offices [2,4].

#### 3.1.3. PM_10_ and PM_2.5_ Concentrations

Burning moxa wools had an influence on the concentrations of PM_2.5_ and PM_10_. Concentrations of PM_2.5_ were in the range of 24–414 μg/m^3^ and those of PM10 were 136–549 μg/m^3^. The average of PM_2.5_ concentrations during the background stage was 26 μg/m^3^ and that during the moxibustion stage was 296 μg/m^3^. The average of PM_10_ concentrations during the background stage was 148 μg/m^3^ and that during the moxibustion stage was 409 μg/m^3^. Both PM_2.5_ and PM_10_ levels in the acupuncture and moxibustion room were higher than the standards stipulated in Taiwan’s Indoor Air Quality Act, which are 35 and 75 μg/m^3^, respectively (8-h average). There is evidence that short-term exposure to PM_10_ has the effects on respiratory health and long-term exposure to PM_2.5_ has a stronger risk than that to PM_10_. It is estimated that all-cause daily mortality increases in the range of 0.2%–0.6% per 10 µg/m^3^ of PM_10_, and cardiopulmonary mortality increases by 6%–13% per 10 µg/m^3^ of long-term exposure to PM_2.5_ [13].

#### 3.1.4. HCHO Concentration

HCHO concentrations increased sharply from the background level of 0.023 ppm (29 μg/m^3^) to a maximum concentration of 0.063 ppm (79 μg/m^3^) when moxibustion started, slowly declining to 0.043 ppm (54 μg/m^3^) at the end of the decay period. Hsu *et al.* investigated the indoor airborne aldehyde emissions in four CMCs during moxibustion and found that mean values of indoor air HCHO were 654 μg/m^3^ (0.52 ppm) in therapy rooms and 155 μg/m^3^ (0.12 ppm) in waiting rooms [10]. In the present study, measured HCHO concentrations were in the range of 0.023–0.063 ppm (28–79 μg/m^3^), which is considerably less than the results reported by Hsu *et al.* and the standard level of 0.08 (100 μg/m^3^) ppm in Taiwan’s Indoor Air Quality Act (1-h average). The reason for the relatively low HCHO levels in this study is that we tested one moxa wool each test time without an accumulative effect of emissions from other moxa wools burned simultaneously and persistently for several accupoints of lots of patients in a real working day. It is a limitation in this study that we did not assess the actual exposure under the usual conditions of moxibustion therapy because the field sampling could cause concerns to the patients In particular, these patients are more sensitive to risk factors of diseases than other people, so it was difficult to convince hospital executives to let us conduct an exposure assessment while patients were receiving moxibustion therapy.

### 3.2. Indoor Air Pollution Control Strategies

#### 3.2.1. Alternative Old Moxa Wool

Mean levels of moisture, ash, and volatile matter in fresh moxa wools were 5.98%, 19.53%, and 74.57%, respectively, by comparison, those in the aged moxa wools were 9.92%, 6.80%, and 83.27%, respectively. After combustion at 850 °C, fresh moxa wools were transformed into white and smaller particulates, but old moxa wools were converted into gray and coarse particulates. Old moxa wools contained more volatile matter and less ash than did fresh moxa wools because they retained more leaf fibers without adding any chemicals or moxa powder during manufacture. Conversely, a part of the leaves was removed from and smoke suppressants as well as moxa powder were added to fresh moxa wools during manufacture, resulting in relatively less volatile matter and lots of ash. Burning rates for fresh and old moxa wools are 0.31 g/min and 0.23 g/min, respectively. Fresh moxa wools had a faster burning rate that did old moxa wools.

**Figure 4:**
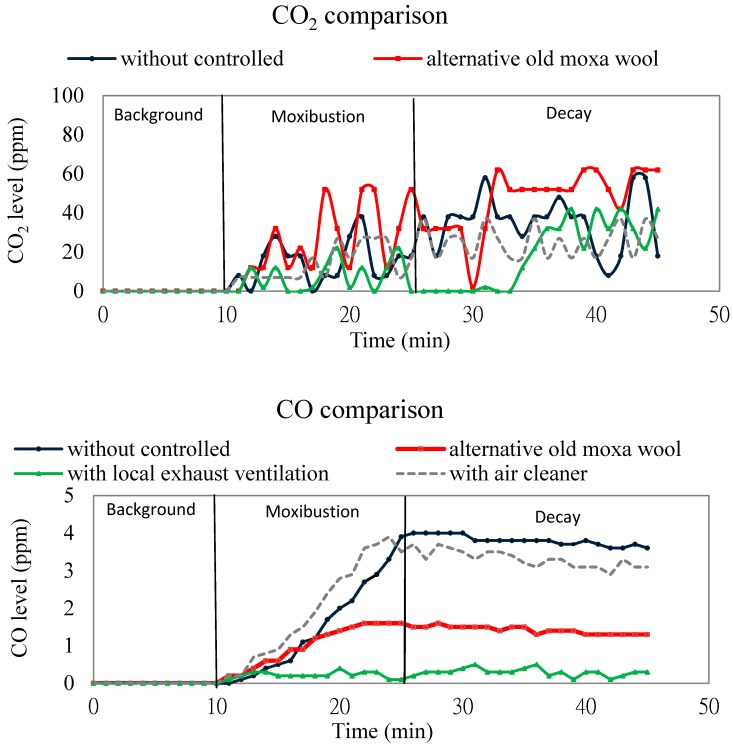

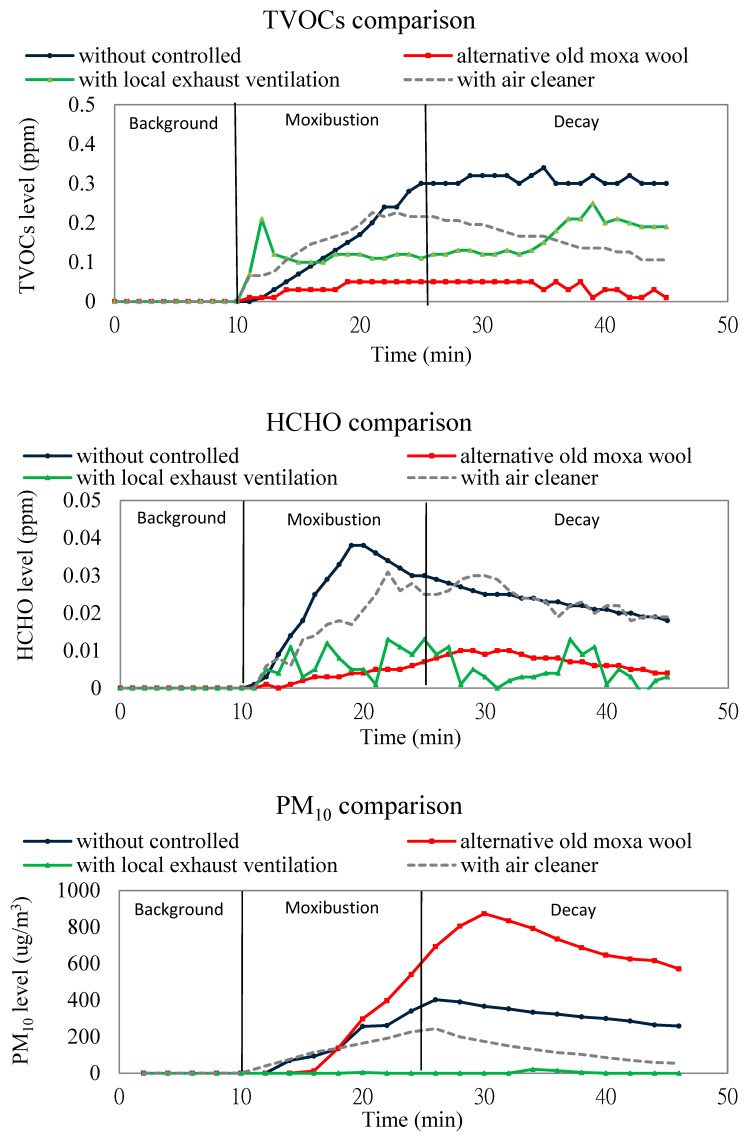

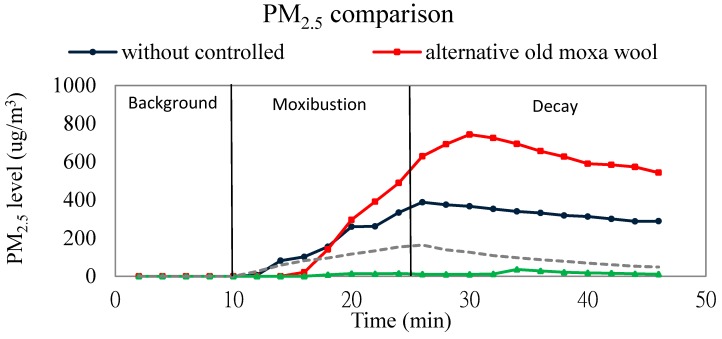
Comparison among the increasing measurements of indoor air pollutants from moxibustion emission inside the acupuncture and moxibustion room.

In this study, when an old moxa wool was burned, CO_2_ concentrations exhibited a similar trend to that observed during burning fresh moxa wool, with an average value of 560 ppm during the background stage and 598 ppm during the moxibustion stage. CO_2_ concentrations slightly increased comparing burning an alternative old moxa wool with a fresh one. CO, TVOC, and HCHO mean concentrations obviously improved when an old moxa wool was replaced. By contrast, PM_10_ and PM_2.5_ concentrations were elevated substantially (Figure 4).

In this study, the CO_2_ concentration increased when old moxa wools were used and the percentage increase of CO_2_ was 41%. By contrast, CO, TVOC and HCHO concentrations differed substantially during burning new and old moxa wools. The average value of CO concentrations during the background and moxibustion stages when burning old moxa wools were <0.1 and 1.4 ppm, respectively. Compared with burning a fresh moxa wool, the CO concentrations decreased by 57%. The combustion of a fresh moxa wool likely produced a large amount of CO because of incomplete combustion. Compared with burning a fresh moxa wool, the emissions of HCHO and TVOCs decreased by 75% and 83%, respectively, possibly because for an old moxa wool the prolonged exposure to air and sunlight during the manufacturing process results in volatilization of some of the volatile oil. However, burning an old moxa wool produced more PM_10_ and PM_2.5_ than did burning a fresh moxa wool. Compared with the emissions while burning new moxa wools, PM_10_ and PM_2.5_ exhibited 1.94- and 1.75-fold increases, respectively, possibly because calcium carbonate, sodium chloride, and other compounds were added to new moxa wools to improve their combustion efficiency in the manufacturing process and these further increased the incomplete combustion (Table 1).

In the qualitative analysis, benzene, toluene, ethylbenzene, styrene, 2-methylfuran, 1-octene, acetone and 1-hexene were found in the emissions from burning fresh moxa wools; while benzene, toluene, ethylbenzene, styrene, 1-decene, D-limonene, phenol, 1-heptene and *p*-xylene existed in old ones. Benzene, toluene, ethylbenzene and styrene were the compounds existing in the emissions from combustion of both fresh and old moxa wools. In the quantitative analysis, the concentrations of benzene, toluene, ethylbenzene and styrene in the emissions from moxibustion of fresh moxa wools were 0.280, 0.178, <0.038 and 0.042 ppm, and those from moxibustion of old ones were 0.068, 0.095, <0.039 as well as <0.034 ppm, respectively. The average TVOC values during the sampling periods for combustion of fresh and old moxa wools were separately 0.93 ppm, ranging from 0.01 to 1.72 ppm, and 0.37 ppm, ranging from 0.02 to 1.10 ppm.

#### 3.2.2. Local Exhaust Ventilation

Before moxibustion, the average values of background mean levels were 555 ppm for CO_2_, 0.1 ppm for CO, 0.026 ppm for HCHO, <0.1 ppm for the TVOCs, and 15 μg/m^3^ for the PM_2.5_ and 49 μg/m^3^ for PM_10_ (Figure 3 and Table 2). Compared with burning a fresh moxa wool without local exhaust ventilation, the emissions decreased by 70% (from 27 ppm to 8 ppm) for CO_2_, 91% (from 2.8 ppm to 0.3 ppm) for CO, 79% (from 0.024 ppm to 0.005 ppm) for HCHO, and 41% (from 0.24 ppm to 0.14 ppm) for TVOCs when the local exhaust ventilation was used. The fact that emissions for PM_2.5_ and PM_10_ respectively decreased by 96% (from 270 μg/m^3^ to 9 μg/m^3^) and 100% (from 262 μg/m^3^ to—6 μg/m^3^) illustrated that the local exhaust ventilation has high efficiency for removing particulates released from moxibustion (Figure 4 and Table 2).

#### 3.2.3. Air Cleaner

Before moxibustion, the background mean levels were 542 ppm for CO_2_, less than 0.1 ppm for CO, 0.025 ppm for HCHO, 0.02 ppm for TVOCs, 6 μg/m^3^ for PM_2.5_, and 39 μg/m^3^ for PM_10_ when the air cleaner was used (Figure 3 and Table 3). Compared with burning a fresh moxa wool without using an air cleaner, the emissions decreased by 22% (from 27 ppm to 21 ppm) for CO_2_, 4% (from 2.8 ppm to 2.7 ppm) for CO, 13% (from 0.024 ppm to 0.021 ppm) for HCHO, and 35% (from 0.24 ppm to 0.16 ppm) for TVOCs when an air cleaner was used. This demonstrates that a light photocatalyst maybe not the best method for controlling gaseous pollutants (Figure 4 and Table 3).

The emissions of PM_2.5_ and PM_10_ were respectively decreased by 71% (from 270 μg/m^3^ to 79 μg/m^3^) and 60% (from 262 μg/m^3^ to 104 μg/m^3^), compared with burning a fresh moxa wool without air cleaner, showing that although the air cleaner can remove more than half of the particulates released from moxibustion, PM_2.5_ and PM_10_ levels remained higher than the standards in the Taiwan Indoor Air Quality Management Act of 35 and 75 μg/m^3^, respectively (8-h average). Although the air cleaner with a HEPA air filter combined with an air ion generator can remove particulates released from moxibustion, however, it seems still has less efficient than local exhaust ventilation.

## 4. Conclusions

In this study, we observed that moxibustion generated numerous air pollutants. Compared with other methods for controlling indoor air pollution, using old moxa wools for moxibustion obviously reduced the gaseous pollutants, namely the CO, HCHO, and TVOCs. Conversely, moxibustion with old moxa wools generated aerosols, such as PM_10_, and PM_2.5_, in concentrations considerably higher than the standards stipulated in the Taiwan Indoor Air Quality Act. Consequently, people in the acupuncture and moxibustion room during moxibustion may be exposed to high concentrations of indoor air pollutants that could elevate their health risk. Using local exhaust ventilation appears to be an effective strategy for controlling indoor air pollutants generated by moxibustion, especially for particulate pollutants. However, it is doubtful that this control strategy would work as well when 20 or more patients per day are receiving moxibustion therapy. Further study with an experimental design that allows parameterization to be determined to enable modeling under a range of conditions should be conducted. Air cleaners are the most common strategies that people use to control indoor air pollution, however, the air cleaner used in the present study was inefficient for controlling the severe levels of indoor air pollutants, particularly gaseous pollutants. Combining old moxa wools and local exhaust ventilation may be suitable for controlling indoor air pollution during moxibustion therapy.

## Figures and Tables

**Table 1:** Comparison between average values of mean levels of air pollutants emitted from burning new moxa wools and alternate old moxa wools.

Pollutant	Stage	Moxa Wools		Percentage Change ^a^
		Fresh	Alternative Old One	
CO_2_	Background level	542 ppm	560 ppm	↑41%
	Moxibustion level	569 ppm	598 ppm	
	Increase level	27 ppm	38 ppm	
CO	Background level	<0.1 ppm	<0.1 ppm	↓57%
	Moxibustion level	2.8 ppm	1.4 ppm	
	Increase level	2.8 ppm	1.2 ppm	
HCHO	Background level	0.025 ppm	0.022 ppm	↓75%
	Moxibustion level	0.049 ppm	0.028 ppm	
	Increase level	0.024 ppm	0.006 ppm	
TVOC	Background level	0.03 ppm	0.03 ppm	↓83%
	Moxibustion level	0.27 ppm	0.07 ppm	
	Increase level	0.24 ppm	0.04 ppm	
PM_2.5_	Background level	26 μg/m^3^	24 μg/m^3^	↑75%
	Moxibustion level	296 μg/m^3^	497 μg/m^3^	
	Increase level	270 μg/m^3^	473 μg/m^3^	
PM_10_	Background level	147 μg/m^3^	198 μg/m^3^	↑94%
	Moxibustion level	409 μg/m^3^	707 μg/m^3^	
	Increase level	262 μg/m^3^	509 μg/m^3^	

**^a^** Percentage change = (Increased mean level of test for moxibustion with old moxa wools—increased mean level of test for moxibustion with new moxa wools)/increase level of test for new moxa wools × 100%.

**Table 2:** Comparison between average values of mean levels of air pollutants emitted from burning new moxa wools with and without local exhaust ventilation.

Pollutant	Stage	Local Exhaust Ventilation		Percentage Change ^a^
		Without Using	Using	
CO_2_	Background level	542 ppm	555 ppm	↓70%
	Moxibustion level	569 ppm	563 ppm	
	Increase level	27 ppm	8 ppm	
CO	Background level	<0.1 ppm	0.1 ppm	↓91%
	Moxibustion level	2.8 ppm	0.4 ppm	
	Increase level	2.8 ppm	0.3 ppm	
HCHO	Background level	0.025 ppm	0.026 ppm	↓79%
	Moxibustion level	0.049 ppm	0.031 ppm	
	Increase level	0.024 ppm	0.005 ppm	
TVOC	Background level	0.03 ppm	<0.1 ppm	↓41%
	Moxibustion level	0.27 ppm	0.14 ppm	
	Increase level	0.24 ppm	0.14 ppm	
PM_2.5_	Background level	26 μg/m^3^	15 μg/m^3^	↓96%
	Moxibustion level	296 μg/m^3^	24 μg/m^3^	
	Increase level	270 μg/m^3^	9 μg/m^3^	
PM_10_	Background level	147 μg/m^3^	49 μg/m^3^	↓100%
	Moxibustion level	409 μg/m^3^	43 μg/m^3^	
	Increase level	262 μg/m^3^	−6 μg/m^3^	

**^a^** Percentage change = (Increase mean level of test for burning new moxa wools using local exhaust ventilation—increase mean level of test for burning new moxa wools without using local exhaust ventilation)/increasing mean level of test for burning new moxa wools without using local exhaust ventilation × 100%.

**Table 3:** Comparison between average values of mean levels of air pollutants emitted from burning new moxa wools with and without air cleaner.

Pollutant	Stage	Air Cleaner		Percentage Change ^a^
		Without Using	Using	
CO_2_	Background level	542 ppm	542 ppm	↓22%
	Moxibustion level	569 ppm	563 ppm	
	Increase level	27 ppm	21 ppm	
CO	Background level	<0.1 ppm	<0.1 ppm	↓4%
	Moxibustion level	2.8 ppm	2.8 ppm	
	Increase level	2.8 ppm	2.7 ppm	
HCHO	Background level	0.025 ppm	0.025 ppm	↓13%
	Moxibustion level	0.049 ppm	0.046 ppm	
	Increase level	0.024 ppm	0.021 ppm	
TVOC	Background level	0.03 ppm	0.02 ppm	↓35%
	Moxibustion level	0.27 ppm	0.18 ppm	
	Increase level	0.24 ppm	0.16 ppm	
PM_2.5_	Background level	26 μg/m^3^	6 μg/m^3^	↓71%
	Moxibustion level	296 μg/m^3^	85 μg/m^3^	
	Increase level	270 μg/m^3^	79 μg/m^3^	
PM_10_	Background level	147 μg/m^3^	39 μg/m^3^	↓60%
	Moxibustion level	409 μg/m^3^	143 μg/m^3^	
	Increase level	262 μg/m^3^	104 μg/m^3^	

**^a^** Percentage change = (Increase mean level of test for burning new moxa wools using air cleaner—increase mean level of test for burning new moxa wools without using air cleaner)/increase mean level of test for burning new moxa wools without using air cleaner × 100%.
